# A Systematic Review on the Effect of Nutraceuticals on Antidepressant-Induced Sexual Dysfunctions: From Basic Principles to Clinical Applications

**DOI:** 10.3390/cimb44080230

**Published:** 2022-07-25

**Authors:** Carmen Concerto, Alessandro Rodolico, Valeria Meo, Donatella Chiappetta, Marina Bonelli, Ludovico Mineo, Giulia Saitta, Sebastiano Stuto, Maria Salvina Signorelli, Antonino Petralia, Giuseppe Lanza, Eugenio Aguglia

**Affiliations:** 1 Psychiatry Unit, Department of Clinical and Experimental Medicine, University of Catania, Via Santa Sofia 78, 95123 Catania, Italy; c.concerto@policlinico.unict.it (C.C.); alessandro.rodolico@phd.unict.it (A.R.); valeriameo26291@gmail.com (V.M.); donatellachiappetta@libero.it (D.C.); marina.bonelli@yahoo.it (M.B.); ludwig.mineo@gmail.com (L.M.); giulia.saitta.91@gmail.com (G.S.); sebastiano.stuto@gmail.com (S.S.); maria.signorelli@unict.it (M.S.S.); petralia@unict.it (A.P.); eugenio.aguglia@unict.it (E.A.); 2 Department of Surgery and Medical-Surgical Specialties, University of Catania, Via Santa Sofia 78, 95123 Catania, Italy; 3 Clinical Neurophysiology Research Unit, Oasi Research Institute-IRCCS, Via Conte Ruggero 73, 94018 Troina, Italy; 4 CERNUT-Research Centre for Nutraceuticals and Health Products, University of Catania, Viale A. Doria 6, 95125 Catania, Italy

**Keywords:** nutraceuticals, sexual dysfunctions, antidepressants, depression

## Abstract

Sexual dysfunctions are common side effects reported by patients during antidepressant treatment. When they occur, patients often discontinue psychopharmacological therapy, with a negative impact on the underlying psychiatric disease. Recently, great attention has been paid to the use of nutraceuticals in the management of psychiatric disorders, although a systematic review on their effects as a treatment option for antidepressant-induced sexual dysfunctions (AISD) is lacking. Here, we conducted a systematic search in the following databases: MEDLINE (through PubMed), EMBASE, PsycINFO, Cochrane Central Register of Controlled Trials, and Web of Science. We searched eligible studies among parallel or crossover randomized controlled trials (RCTs) in adult populations. After this process, a total of 10 articles that evaluated the effect of six different nutraceuticals versus placebo were included: Maca Root, S-adenosyl-L-methionine (SAMe), Rosa Damascena, Ginkgo Biloba, Saffron, and Yohimbine. Overall, a high dose of Maca Root and the use of SAMe or Saffron may improve AISD. Additionally, the administration of Rosa Damascena seemed to be more effective in men than in women, whereas no evidence of effects emerged for Gingko Biloba and Yohimbine. Given the mixed results still available, future RCTs should consider larger samples and confounding factors, such as depressive status and individual vulnerability.

## 1. Introduction

Sexual dysfunctions (SDs) are one of the most frequent and distressing side effects experienced by patients taking antidepressants (ADs), including tricyclic antidepressants (TCAs), monoamine oxidase inhibitors (iMAO), selective serotonin reuptake inhibitors (SSRI), and dual noradrenergic/serotonergic reuptake inhibitors (SNRI) [1,2]. According to previous studies, SDs are reported by 25% up to 75% of patients taking SSRIs and by 30% up to 93% of those taking TCAs [1,3]. SDs may engage the phase of the sex drive (loss of libido), the arousal (lubrication in women, erectile function in men), the ejaculation, and orgasm [4]. Data collected in recent decades confirm that there are significant differences in antidepressant-induced SD (AISD) between men and women. Accordingly, the side effects most reported by women are reduced sexual desire and sexual arousal, as well as difficulties in achieving orgasm [5]. Conversely, men taking serotonergic drugs often complained of ejaculatory delay, whereas those taking TCAs and iMAO mainly reported impaired orgasm achievement [6].

It has been hypothesized that one of the mechanisms underlying AISD might be related to an overactivation of the post-synaptic 5-hydroxytryptamine (5-HT)_2_A receptors in the raphe nuclei of the midbrain. Indeed, neurons in that area inhibit dopamine release in the hypothalamus, in the mesolimbic system, and in cortical areas which are responsible for processing the motivational, autonomous, and emotional components of the sexual act [7]. Moreover, those neurons inhibit peripheral nerves which regulate the function of genitalia, thus altering the mechanistic aspects of sexual functioning [8]. Not surprisingly, SSRIs and SNRIs are the ADs most associated with sexual adverse effects; in particular, escitalopram and paroxetine have a higher risk of SD than other antidepressants [1].

Varieties of interventions have been suggested for the management of AISD. First of all, to verify whether these side effects are reversible, clinicians might consider waiting for few weeks for spontaneous symptom remission. Alternatively, clinicians can opt for specific pharmacological interventions, such as the use of phosphodiesterase-5 inhibitors, which are usually effective in the treatment of AD-induced erectile dysfunction and anorgasmia in men [9]. Other suggestions include the prescription of the lowest effective dose of AD, the switch to another class of AD, and the so-called “weekend holiday”, in which patients do not intake ADs 24–48 h before the sexual intercourse [6,10]. However, despite the aforementioned clinical strategies and the interventions proposed, clear guidelines for the management of these side effects are still lacking [9]. Moreover, the large number of people taking ADs worldwide, the use of ADs for several psychiatric disorders [11,12,13,14,15], and the potential adverse impact on treatment compliance and long-term outcome calls for broadening the range of approaches to AISD treatment.

In this scenario, the Complementary and Alternative Medicine (CAM) [16], a broad and heterogeneous group of therapeutic approaches including natural supplements, plant-based phytoceuticals [17,18,19], and nutrient-based nutraceuticals [16], seems to enhance human health and may have a positive impact on a variety of diseases [20,21,22,23]. Recently, attention has been given to nutraceuticals, defined as a “natural substance such as food or a part of them, a vitamin, a mineral, or a herb with beneficial effects for human health”, administered alone or in add-on therapy to standard care [24]. Nutraceuticals have also been given significant attention as an alternative therapeutic approach because they are considered more affordable and widely available [16]. As such, over the last two decades, interest and demand for nutraceuticals have grown worldwide, and the increased interest in finding novel therapeutic options to treat SDs has led to a consistent increase in the use of nutraceuticals [9,25]. Namely, Maca Root [26,27,28], Saffron [29], S-adenosyl-L-methionine (SAMe), Ginkgo Biloba [30,31], and Rosa Damascema [32,33] have been used for several illnesses, including SDs.

However, a systematic review on the effects of these nutraceuticals on AISD is currently lacking. Given the growing clinical and scientific interest to this field of research, we systematically reviewed the existing literature on the effects of nutraceuticals for the treatment of AISD in adult populations.

## 2. Materials and Methods

### 2.1. Literature Search

This systematic review was planned and conducted in accordance with the PRISMA guidelines [34]. On 20 November 2021, we performed a systematic search in the following databases: MEDLINE (through PubMed), EMBASE, PsycINFO, Cochrane Central Register of Controlled Trials, and Web of Science. The following search string were used: ((“Dyspareunia” OR “Penile erection” OR “orgasm” OR “priap*” OR “lubricat*” OR “sexual dysfunct*” OR “sexual problem*” OR “sexual arousal” OR “sexual satisf*”) AND ((agomelatine OR amisulpride OR amitriptyline OR amoxapine OR atomoxetine OR bupropion OR buspirone OR citalopram OR clomipramine OR desipramine OR desvenlafaxine OR dothiepin OR doxepin OR duloxetine OR escitalopram OR fluoxetine OR fluvoxamine OR imipramine OR isocarboxazid OR ketamine OR lofepramine OR maprotiline OR mianserin OR milnacipran OR mirtazapine OR moclobemide OR nefazodone OR nortriptyline OR paroxetine OR phenelzine OR protriptyline OR reboxetine OR selegiline OR sertraline OR sulpride OR tianeptine OR tranylcypromine OR trazodone OR trimipramine OR venlafaxine OR vilazodone OR vortioxetine) OR (ssri* OR snri* OR ndri* OR NRI* OR sari* OR maoi* OR tricyclic* OR antidepress*)) AND ((“randomized controlled trial” OR “controlled clinical trial” OR RCT OR randomized OR placebo OR “drug therapy” OR randomly OR trial OR groups) NOT (animals NOT humans))). We extended the abovementioned search until 31 May 2022.

### 2.2. Eligibility Criteria

Eligibility criteria were: (1) parallel or crossover randomized controlled trials (RCTs); (2) studies comparing CAM compounds to each other, or versus placebo, or versus pharmacological compounds on sexual side effects concomitant to treatment with ADs; (3) studies in adult populations, over the age of 18; (4) English-written studies. The exclusion criteria were: (1) quasi-randomized studies (e.g., alternation or methods based on admission dates); (2) non-peer reviewed data (e.g., abstracts or trial registries repositories); (3) preclinical studies on in vitro experiments; (4) any other publication different from research studies (e.g., review, case report, commentary, editorial).

### 2.3. Data Extraction

Two independent reviewers (V.M. and A.R.) initially screened each title and abstract of the articles retrieved, and then selected the studies to be included, after checking the full text. In case of disagreement, the final decision was made with the help of a third reviewer (C.C.). Subsequently, relevant data were extracted in a predefined form. The following variables were collected: first, author and year of publication, country where the study was conducted, study design, recruitment time, follow-up duration, study population characteristics, study aim, intervention description and main results (both in terms of efficacy and tolerability). After double-checking the extracted data, the resulting forms were merged in a comprehensive table. Meta-analysis was not planned due to the paucity of trials available and the heterogeneity of available compounds. Quality assessment was performed by using the version 2.0 of the Cochrane Risk of Bias tool. Thereafter, we selected the SDs as the outcome of choice. The review protocol can be found on INPLASY Platform (https://inplasy.com/inplasy-2021-11-0051, accessed on 6 July 2022).

## 3. Results

**Figure 1:**
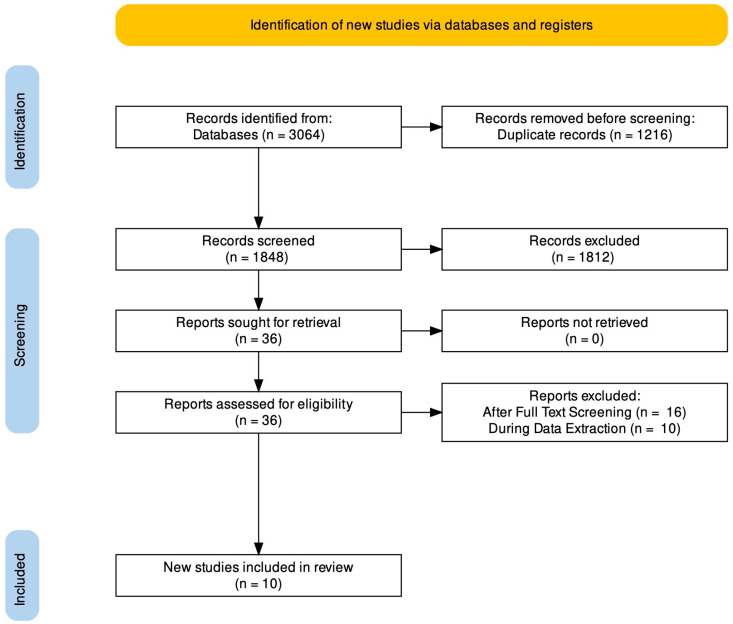
PRISMA 2020 flow diagram showing the search strategy, the number of records identified, and the number of included/excluded studies.

The PRISMA flow chart is shown in Figure 1. A total of 3064 potentially relevant studies were identified. After the deduplication procedure, 1848 records remained. After screening titles and abstracts, 1812 records were excluded. Among the remaining 36 records, only 10 full text articles evaluating the effect of six different nutraceuticals (Maca Root, SAMe, Rosa Damascena, Ginkgo Biloba, Saffron, and Yohimbine) versus placebo were considered eligible according to the inclusion/exclusion criteria, and therefore included. These studies were published between 2002 and 2019 and carried out in the USA (n = 4), Iran (n = 4), South Korea (n = 1), and UK (n = 1). All the studies were RCTs, 9 double-blinded [35,36,37,38,39,40,41,42,43] and 1 triple-blinded [44], and they assessed a population of depressed patients treated with ADs and suffering from AISD. One study compared different doses of Maca Root [35], one study Maca Root and placebo [37], one SAMe and placebo [36], two Rosa Damascena and placebo in males and females, respectively [38,39], two Gingko Bilboa and placebo [40,44], two Saffron and placebo [41], and one Yohimbine (along with Olanzapine and Mirtazapine) and placebo [42]. Table 1 shows a detailed summary of the core characteristics of the studies included. 

### 3.1. Maca Root

Dording et al. [35] conducted a double blind, randomized parallel group study to investigate the effect of high (1.5 g/day) and low (3.0 g/day) dose of Maca Root intake on AISD. Subjects were 20 remitted depressed patients on a stable dose of ADs for at least 4 weeks. SDs were measured with the Arizona Sexual Experience Scale (ASEX) and the Massachusetts General Hospital Sexual Function Questionnaire (MGH-SFQ). Patients mostly complained of delayed orgasm, difficulty with arousal, and lack of libido. Ten subjects completed the study and 16 subjects were eligible for an intention to treat (ITT) analysis. ITT subjects on the high dose showed a significant improvement in ASEX and MGH-SFQ scales, meanwhile libido improved for all subjects. The degree of improvement appeared to be dose related. A significant improvement in the 17-item Hamilton Depression Rating Scale scores for the ITT recipients of the high-dose was also reported.

In another RCT by Dording et al. [37], treatment with Maca Root (150 mg bid, 12 weeks) was explored in 42 remitted depressed women taking a stable dose of ADs and with AISD. Interestingly, the authors conducted a subgroup analysis based upon menopausal status. There was not a significant difference between the Maca group and the placebo group in total ASEX and MGH-SFQ scores, either overall or within premenopausal or postmenopausal subgroups. Furthermore, the authors explored the correlation between scale score and testosterone level: patients in the Maca group who showed improvement in the ASEX score demonstrated a greater change in testosterone concentrations.

### 3.2. SAMe

Adjunctive SAMe treatment for SDs has been evaluated by Dording et al. [36]. The authors explored the effect of SAMe supplementation on SDs by using the MGH-SFQ in a group of non-responder patients to SSRI/SNRI medications. A total of 55 patients completed the 6-week study. The results showed that adjunctive SAMe (800 mg, 6 weeks) was beneficial to sexual functioning in men, especially arousal and erection.

### 3.3. Rosa Damascena

Farnia et al. conducted two RCTs [38,39] investigating the use of Rosa Damascema oil on SSRI-induced SD. The first RCT investigated its effect on SD assessed with the Brief Sexual Function Inventory (BSFI) in a group of male patients suffering from acute depressive state and AISD. Sexual function significantly improved over time, and a significant correlation between depressive symptoms and SD was observed in the placebo group. The second RCT involved 50 depressed women with SSRI-induced SDs explored with the Female Sexual Function Index (FSFI). The authors evaluated the effect of Rosa Damascena oil augmentation versus placebo for 8 weeks. The results showed a modest effect on female SDs. Sexual desire, orgasms, and satisfaction significantly increased in both treatment groups, although without statistical significance between groups.

### 3.4. Ginkgo Biloba

Two RCTs explored the effect of Ginkgo biloba on AISD. In the study by Kang et al. [40], 24 patients were randomized to adjunctive Ginkgo Biloba or placebo for 12 weeks. At weeks 2, 4, and 8 after treatment, there was no statistically significant difference between the two groups. In comparison to baseline, both the Ginkgo Biloba group and the placebo group showed improvement in some phases of the sexual response cycle, although evaluated with an unvalidated questionnaire. Similarly, the triple-blind RCT by Wheatley et al. [44] reported no significant difference between treated and placebo.

### 3.5. Saffron

Modabbernia et al. [43] explored the efficacy of Saffron on fluoxetine-induced SD. Namely, 36 depressed male patients were randomized to either Saffron or placebo for 4 weeks. The International Index of Erectile Function scale (IIEF) was used to assess sexual function at baseline and at weeks 2 and 4. By week 4, Saffron resulted in a significantly greater improvement in sexual functions (erectile function and intercourse satisfaction domains) compared to placebo. There was also a trend toward improvement in orgasmic function, overall satisfaction, and sexual desire in Saffron-treated patients.

These findings were similar to those obtained by Kashani et al. [41], who investigated the safety and efficacy of Saffron on fluoxetine-induced SD in 34 women. Remitted depressed women were randomized to adjunctive Saffron or placebo for 4 weeks. At the end of the study, patients in the Saffron group showed significantly greater improvement in total FSFI, as well as arousal, lubrication, and pain domains compared to placebo, but not in desire, satisfaction, and orgasm domains. Desire, satisfaction, and orgasms subscales trended higher in the Saffron group, but not significantly different from placebo.

### 3.6. Yohimbine

The study by Michelson et al. [42] compared the effects of yohimbine with those of two non-nutraceuticals compounds (mirtazapine and olanzapine), all versus placebo, for 6 weeks in 148 premenopausal women with fluoxetine-induced SD. The results did not reveal statistically significant differences among interventions.

### 3.7. Quality of the Studies Included

**Figure 2:**
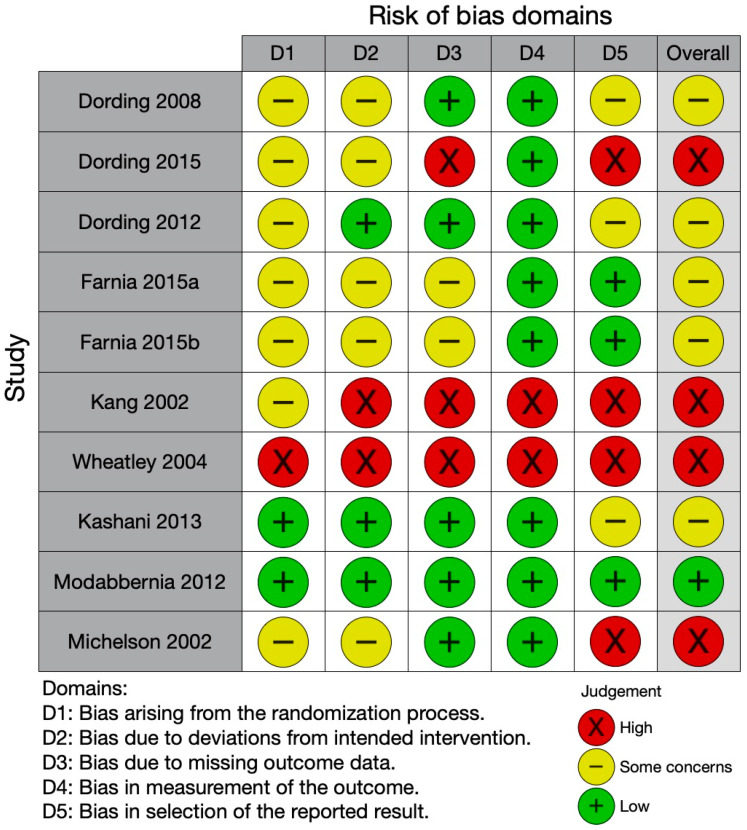
Quality assessment of the included studies [35,36,37,38,39,40,41,42,43,44].

As shown in Figure 2, the quality assessment of the included studies resulted in an overall moderate or poor quality, except for the study by Modabbernia et al. [43], comparing Saffron and placebo in males, that was at low risk of bias. The randomization process also arose some concerns for seven studies: it was at high risk for one study and at low risk for two studies. The risk of bias due to deviations from intended intervention was low for three studies only, which accounted for missing data. We defined at risk of bias for missing data those studies with 5% missing data for SD, as suggested by the Cochrane RoB 2.0, unless any corrective statistical procedure was applied, or the dropout rate was equally distributed between groups. Half of the studies were at low risk of bias for missing data, while the remaining were at high risk or aroused some concerns. Most of the studies used validated clinical scales for measuring sexual side effects, except for those by Kang et al. [40] and by Wheatly et al. [44] Finally, only part of the trials had a trial registration allowing us to check for selection bias. Three studies only complied with their trial registration, and we considered them at low risk of bias; for the other studies, the trial registration was missing, or the study deviated from it, if present.

## 4. Discussion

### 4.1. Summary of the Evidence and Proposed Mechanisms

To the best of our knowledge, the present article is the first systematic review of RCTs examining the effect of nutraceuticals on AISD in an adult population. Overall, the results allow us to conclude that Maca Root, SAMe, Rosa Damascena, and Saffron may be effective in a variety of sexual function domains.

In further detail, the RCTs evaluating the effect of Maca showed positive results. As known, Maca preparations are obtained from the Andine plant Lepidium meyenii and are traditionally used for libido and fertility enhancement [45]. In the study by Dording et al. [35], all subjects showed improvement in sexual function and most of the patients were women. It has been hypothesized that Maca Root might improve libido and enhance spermatogenesis, as these effects were ascribed to some new discovered metabolites, such as “macamides” [46,47]. The significant improvement in libido supports previous findings in animal studies, although they have not been confirmed due to the inconclusive results produced by the in vivo replication [45]. Conversely, the positive effect of Maca Root on AISD was confirmed by another study by Dording et al. [37] on women patients. The authors reported a significant correlation between change in testosterone level and sexual functions improvement, steered by the postmenopausal women subgroup. A positive effect might be hypothesized regarding Maca on menopausal symptoms and hormone levels in women. Accordingly, a previous study on postmenopausal women treated with Maca showed a significant decrease in FSH level [48]. Therefore, in women, it might be hypothesized that Maca may have an indirect androgenic response through a negative feedback pathway involving the LH/FSH, whereas the increased production of androgens might explain the improvement in sexual functioning. Taken together, these findings may shed light on the mechanisms of Maca, although previous findings in men failed to prove a direct androgenic effect [49,50]. Probably, the belief that Maca can restore hormonal balance in menopause is undermined by the relatively small sample size and the significant positive placebo effect reported in all studies [45]. Nevertheless, there is no evidence for an androgen-mediated action of Maca, and its site of action (central or peripheral) has not yet been identified.

The other nutraceutical reported to exert an effect on AISD is SAMe, which is the principal biological methyl donor found in all living organisms [51]. SAMe improved both arousal and erectile dysfunction in men compared to placebo, regardless of the severity of depressive symptoms [36]. It has been suggested that SAMe may cause vasodilation and relaxation of the corporal smooth muscle, thus facilitating erectile function, likely through the nitric oxide signaling [52], although systematic studies are needed.

Rosa Damascena intake showed an overall positive affect on AISD in men [39]. Known as Damask rose, it is a plant used for centuries for a variety of diseases [33], albeit the underlying mechanism of action is not fully understood yet. It is possible that Rosa Damascena oil plays an antagonistic effect on the stimulation of the postsynaptic 5-HT2 and 5-HT3 receptors and an antagonistic effect on cortico-limbic 5-HT receptors, eventually increasing sexual desire and orgasm [33,52,53]. These findings might explain the clinical improvement, although the mechanism of action is still debated.

The two RCTs evaluating the effect of Saffron on AISD revealed its superiority compared to placebo. Saffron (Crocus sativus L.) and its extract are used as a medicinal plant in the traditional medicine [53]. Saffron was effective in treating the arousal and lubrication dysfunctions but was ineffective for other SDs, including desire, satisfaction, and orgasm [41]. In the study by Modabbernia et al. [43], Saffron showed an effect on erectile function and intercourse satisfaction but did not affect other subscales of SDs. Sexual improvement by Saffron might be explained by the effects of crocin and its active components [54]. Recently, some animal and human studies have shown the favorable effects of Saffron, particularly its crocin component, on SD [54,55]. It is known, indeed, that Saffron may interact with nitric oxide and the opioid system, both playing a key role in erectile function [52,56]. Yet, Saffron was not effective in all domains of SD, hence advocating that it might selectively improve sexual behavior.

To summarize, Maca Root, SAMe, Rosa Damascena, and Saffron proved their ability in ameliorating AISD. Regarding the mechanism of action, these nutraceuticals may display a single or a combined mechanism, eventually resulting in enhanced sexual functioning. Different sex-related effects were also noted; these findings were probably related to the non-comparable psychophysiological sexuality pathways of males and females. In addition, some individual vulnerabilities and physiologic variables should be taken into account when considering the response to nutraceuticals. Indeed, it cannot be excluded that these factors, alone or in combination, might have contributed to the lack of evidence for the beneficial effects for the use of Gingko Biloba and Yohimbine.

The availability of an augmentation strategy that improves SD has the potential to largely improve compliance and the quality of life of depressed patients. However, we should consider that patients might complain of SD not only as a side effect of the AD treatment, but also because of their untreated or partially treated depressive illness. Indeed, the importance of evaluating sexual functioning both before and after AD treatment is well known, in order to obtain information about either AISD or improvement due to the amelioration of depressive symptoms.

### 4.2. Conclusions

We systematically reviewed the role of nutraceuticals in AISD. Although some evidence emerged for the beneficial effect of Maca Root, SAMe, Rosa Damascena, and Saffron, the results are still mixed. Future RCTs should include larger samples and consider any possible confounding factor, such as depressive status and individual vulnerability.

## 5. Study Limitations and Research Agenda

This systematic review has some limitations. In general, our results should be viewed in light of the overall low quality of the studies included. Moreover, the heterogeneity of the interventions and instruments included did not allow us to perform any statistical analyses, thus presenting the data in a narrative form. We found few eligible studies. We decided to exclude “grey literature” and to only include rigorous studies, although we are aware that this may have quantitatively reduced the amount of evidence. Finally, it should be noted that most of the included studies have been conducted in Iran and the USA, thus reducing the generalizability of the results.

Future studies on nutraceuticals should report more detailed information about the blindness of investigators, raters, and participants. It is also necessary to predefine clear trial registrations (e.g., a predefined numerosity of patients to be included) by appropriate sample size calculation. Finally, studies should be multicentered and geographically distributed to ensure the adequate generalizability of the results, also accounting for patient dropouts. This research agenda will also allow a meta-analysis of the studies in the future, thus acknowledging a reliable comparison of nutraceutical interventions for AISD.

## Figures and Tables

**Table 1:** Detailed description of the studies included.

Author, Year (Country) Study Design Recruitment Timing Treatment Duration Follow-Up Study Population Characteristics	Interventions	Efficacy	
			Tolerability
***Dording C. M* et al., *2008* [35] *(USA)*** **Design:** double-blind, randomized parallel group **Recruitment Timing:** April 2005–February 2006 **Treatment duration:** 12 weeks **Follow-up:** not reported **Participants.** 20 (10 low dose; 10 high-dose) **Female/Male:** 17/3 **Mean Age (SD):** 36 (13) **Diagnosis:** major depressive disorder **Clinical Status:** remitted depressed outpatients with sexual dysfunctions (SD) **Cause of disfunctions:** selective serotonin reuptake inhibitors (SSRI)-induced SD	***Maca Root (high dose)*** **Dose scheme:** 6 capsules of 500 mg/day **Application:** Pills ***Maca root (low dose)*** **Dose scheme:** 6 capsules of 250 mg/day **Application:** Pills	***Efficacy*** **Measures:** Arizona Sexual Experiences Scale (ASEX) and Massachusetts General Hospital-Sexual Functioning Questionnaire (MGH-SFQ) **Results:** ITT subjects on high dose Maca showed an improvement in ASEX and MGH-SFQ scales Libido improved for both ITT and completers groups.	
		***Tolerability*** Well tolerated overall Adverse effect reported: - Gastrointestinal upset = 5 - Headache = 2 - Irritability = 2 - Panic attack = 1 - Urinary frequency = 1 - Blurry vision = 1 - Sleep disruption = 1 - Increased sweating = 1 - Increased dreaming = 1 - Thicker menstrual discharge = 1	
***Dording C.M.* et al., *2015* [37] *(USA)*** **Design:** double-blind placebo-controlled trial **Recruitment Timing:** December 2007- June 2010 **Treatment duration: 12 weeks** **Follow-up:** 3 months **Participants:** 42 (21 maca; 21 placebo) **Female/Male:** only female **Mean Age (SD):** 41.5812.5) **Diagnosis:** major depressive disorder **Clinical Status:** remitted depressed outpatients with antidepressant induced sexual dysfunction (AISD) **Cause of disfunctions:** SSRI -induced SD	***Maca Root 1500* mg** **Dose scheme:** 1500 mg bid **Application:** not reported ***Placebo*** **Application:** not reported	***Efficacy*** **Measures:** ASEX and MGH-SFQ **Results:** mean change in total ASEX and MGH-SFQ scores was not significantly different for the maca versus the placebo groups, either overall or within premenopausal or postmenopausal subgroups. Change in testosterone level correlated significantly with improvements in sexual functioning in the maca group.	
		***Tolerability*** Three subjects discontinued Adverse events reported: - flu-like symptoms - vomiting	
***Dording, C.M.* et al., *2012* [36] *(USA)*** **Design:** randomized, double-blind trial **Recruitment Timing:** not reported **Treatment duration:** 6 weeks **Follow-up:** not reported **Participants:** 58 (31 SAMe; 27 placebo) **Female/Male:** 34/24 **Mean Age (SD):** SAMe 51(14.2); Placebo 48 (9.7) **Diagnosis:** major depressive disorder **Clinical Status:** SSRI/SNRI non-responders depressed patients **Cause of disfunctions:** SSRI/SNRI-induced SD	***S-adenosyl-L-methionine (SAMe) 800* mg** **Dose scheme:** two 400 mg *SAMe* pills daily for 2 weeks then doubled **Application:** pills ***Placebo*** **Dose Scheme:** two dummy pills daily, each identical to a 400 mg pill in appearance **Application:** pills	***Efficacy*** **Measures:** MGH-SFQ **Results:** men treated with adjunctive SAMe demonstrated significantly lower arousal dysfunction and erectile dysfunction at endpoint than those treated with adjunctive placebo	
		***Tolerability*** Tolerability data not reported	
***Farnia* et al., *2015a* [39] *(Iran)*** **Design:** randomized, double-blind, placebo-controlled clinical trial **Recruitment Timing:** October 2013–June 2014 **Treatment duration:** 8 weeks **Follow-up:** 8 weeks after the study started **Participants:** 60 (30 + 30) **Female/Male:** only male **Mean Age (SD):** Rosa Damascena 32.45 (5.68); Placebo 34.02 (6.45) **Diagnosis:** major depressive disorder (DSM-5 criteria) **Clinical Status:** outpatient with moderate depression in continuous treatment with SSRI who suffer from SSRI-inducted SD **Cause of disfunctions:** SSRI/SNRI-induced SD	***Rosa Damascena*** **Dose scheme:** 2 mL/day (containing 17 mg Citronellol of essential oil of *R. damascena*) **Application:** solution ***Placebo*** **Dose scheme:** 2 mL/day (oil–water solution with an identical scent) **Application:** solution	***Efficacy*** **Measures:** Brief Sexual Function Inventory (BSFI) **Results:** SD improved more in the Rosa Damascena group than in the placebo group. Improvements were observed in the Rosa Damascena group from week 4 to week 8	
		***Tolerability*** Tolerability data not reported	
***Farnia* et al., *2015b* [38] *(Iran)*** **Design:** randomized, double-blind, placebo-controlled clinical trial **Recruitment Timing:** October 2013–June 2014 **Treatment duration:** 8 weeks **Follow-up:** 8 weeks after the study started **Participants:** 50 (25 + 25) **Female/Male:** only female **Mean Age (SD):** 34 years **Diagnosis:** major depressive disorder (DSM-5 criteria) **Clinical Status:** outpatients with acute depressive state stabilized and in continuous treatment with SSRI who suffer from SSRI-inducted SD **Cause of disfunctions:** SSRI -induced SD	***Rosa Damascena*** **Dose scheme:** 2 mL/day (containing 17 mg Citronellol of essential oil of *R. damascena*) **Application:** solution ***Placebo*** **Dose scheme:** 2 mL/day (oil–water solution with an identical scent) **Application:** solution	***Efficacy*** **Measures:** Female Sexual Function Index (FSFI) **Results:** sexual desire, sexual orgasms and sexual satisfaction increased over time. Patients in the Rosa Damascena group reported decreased pain. Overall sexual score increased in the Rosa Damascena as compared to the placebo condition. Data suggest only modest effects of adjuvant Rosa Damascena oil on female sexual function.	
		***Tolerability*** Tolerability data not reported	
***Kang* et al., *2002* [40] *(South Korea)*** **Design:** randomized, placebo-controlled, double-blind trial **Recruitment Timing:** March 2000–January 2002 **Treatment duration:** 2 months **Follow-up:** not reported **Participants:** 37 (19 GB + 18 P) **Female/Male:** 10 (4 GB + 6 P)/27 (15 GB + 12 P) **Mean Age (SD):** 47.31 (10.51) GB–45.50 (8.80) P **Diagnosis:** substance-induced SD due to antidepressants (DSM-IV criteria) in patients with depressive disorder (without psychotic features) or anxiety disorder **Clinical Status:** depressive disorder or anxiety disorder being treated with an antidepressant **Cause of disfunctions:** SSRI and tricyclic-induced SD	***Ginkgo Biloba*** **Dose scheme:** 120 mg daily for the first 2 weeks, 160 mg for the second 2 weeks, 240 mg was dispensed for 4 weeks thereafter. **Application:** pills ***Placebo*** **Dose scheme:** same as above but with placebo pills. **Application:** pills	***Efficacy*** **Measures:** not validated questionnaire **Results:** no statistically significant difference was shown between the two groups; in comparison with baseline, both the Ginkgo biloba group and the placebo group showed improvement in some part of the sexual function, which is suggestive of the importance of the placebo effect in assessing sexual function.	
		***Tolerability*** Well tolerated; only few patients complained of: - GI disturbances - Headache - Sedation - Increased Oral Intake	
***Wheatley D. 2004* [44] *(UK)*** **Design:** triple-blind (investigator, patient, statistician), randomized, placebo-controlled, trial **Recruitment Timing:** not reported **Treatment duration:** 12 weeks **Follow-up:** 18 weeks **Participants:** 24 (13 Placebo–11 Gingko Biloba) **Female/Male:** 5/8 Placebo–5/6 Gingko Biloba **Mean Age (SD):** 40 **Diagnosis:** long standing depression **Clinical Status:** taking any antidepressant drug for at least 2 weeks and experiencing sexual problems **Cause of disfunctions:** SSRI and tricyclic-induced SD	***Gingko Biloba*** **Dose scheme:** 125 mg daily for one week before the study to begin, then 250 mg daily **Application:** pills ***Placebo*** **Dose scheme:** equivalent as above once a day. **Application:** pills	***Efficacy*** **Measures:** Sexual Dysfunction Questionnaire (SDQ) **Results:** there were no significant differences from week 0 at any period of the trial, other than at week 6 in the Ginkgo Biloba group; neither were there any significant between-group differences. There were no significant differences between week 18 and either week 0 or week 12.	
		***Tolerability*** Low incidence of side effects (no side effects for 10, Ginkgo Biloba patients, nor for 11 placebo patients) One severe side effect with consequent dropout in the placebo group (for night sweats) and three in the Ginkgo Biloba group (for gastric pain; ‘muzzy head’; paresthesia and anesthesia of hands with palpitations)	
***Kashani* et al. *2012* [41] *(Iran)*** **Design:** randomized, double-blind, placebo-controlled, parallel-group study conducted in three centers (two private clinics and one hospital outpatient clinic) **Recruitment Timing:** February 2009–February 2010 **Treatment duration:** 4 weeks **Follow-up:** not reported **Participants:** 38 (19 Saffron–19 Placebo) **Female/Male:** only female **Mean Age (SD):** Saffron 34.7 (4.7)–Placebo: 36.0 (6.1) **Diagnosis:** major depressive disorder (DSM IV criteria) **Clinical Status:** non responders to antidepressant + in treatment with fluoxetine **Cause of distinctions:** fluoxetine-induced SD	***Saffron*** **Dose scheme:** 15 mg twice a day or placebo **Application:** pills ***Placebo*** **Dose scheme:** same as above **Application:** pills	***Efficacy*** **Measures:** FSFI **Results:** saffron was particularly effective in improving the arousal, lubrication, and intercourse-related pain domains of FSFI. Saffron was shown to be as safe as placebo	
		***Tolerability*** Well tolerated, no significant differences between the two groups. Side effects reported: - Increased appetite - Dizziness - Sore throat - Decreased appetite - Headache - Insomnia - Sedation - Nausea	
***Modabbernia* et al., *2012* [43] *(Iran)*** **Design:** randomized, double-blind, placebo controlled, parallel-group clinical trial conducted in one hospital outpatient clinic **Recruitment Timing:** February 2009–December 2011 **Treatment duration:** 4 weeks **Follow-up:** not reported **Participants:** 30 (15 saffron + 15 placebo) **Female/Male:** only male **Mean Age (SD):** Saffron 36.6 ± 8.3 Placebo 40.5 ± 9.4 **Diagnosis:** major depressive disorder (DSM IV criteria) **Clinical Status:** SD emerged during treatment and documented with a score < 25 on the erectile function domain of International Index of Erectile Function scale (IIEF) **Cause of distinctions:** fluoxetine-induced SD.	***Saffron*** **Dose scheme:** 15 mg twice per day or placebo (with the same appearance and taste as saffron capsule) twice per day. **Application:** pills ***Placebo*** **Dose scheme:** same as above but with placebo pills twice per day. **Application:** pills	***Efficacy*** **Measures:** International Index of Erectile Function scale (IIEF) **Results:** there was evidence for beneficial effects of saffron on fluoxetine-induced SD. This was particularly evident in the erectile and intercourse satisfaction domains of IIEF There was evidence for tolerability and efficacy of saffron in treatment of fluoxetine-related erectile dysfunction.	
		***Tolerability*** Frequency of side effects did not differ between the two treatment groups. Nine side effects (all mild, none determining dropout): - Abdominal pain - Daytime drowsiness - Nausea - Decreased appetite - Dry mouth - Nervousness - Restlessness - Morning drowsiness - Increased appetite	
***Michelson* et al., *2002* [42] *(USA)*** **Design:** double blind, randomized, parallel, placebo-controlled, multi-site trial **Recruitment Timing:** not reported **Treatment duration:** 10-week study consisting of a 4-week baseline evaluation followed by a 6-week treatment period **Follow-up:** not reported **Participants:** 148 (Yohimbine 35, Placebo 39, non-nutraceuticals: Mirtazapine 36, Olanzapine 38) **Female/Male:** only female **Mean Age (SD):** Yohimbine 35.4 (7.0), Placebo 35.5 (7.0) **Diagnosis:** major depressive disorder + others (not specified) **Clinical Status:** patients treated with a stable dose of fluoxetine with a satisfactory response to treatment and either anorgasmia, a marked decrease in ability to reach orgasm, or impaired vaginal lubrication during sexual activity which must have begun after the initiation of fluoxetine therapy **Cause of distinctions:** SSRI-induced SD	***Yohimbine*** **Dose scheme:** 5.4 mg/day, 1–2 h before the sexual relations. Dose increased to 10.8 mg/day, after 1 week if tolerated. **Application:** pills ***Placebo*** **Dose scheme:** 2.5 mg/ day, 1–2 h before the sexual relations. Dose “doubled” daily, after 1 week, if tolerated. **Application:** pills	***Efficacy*** **Measures:** Kinsey Ratings of Sexual Function—modified (KRSF) **Results:** no drug assessed was consistently associated with differences from placebo. The results of the study do not support uncontrolled reports of efficacy for these agents in premenopausal women.	
		***Tolerability*** Patients treated with yohimbine did not discontinue the treatment more frequently than placebo ones (placebo 5% and yohimbine 11%). Side effects of intervention groups were reported aggregated for all the treatment groups, and those with frequency higher than 10% and statistically significantly different from placebo were: - Somnolence - Insomnia - Weight gain - Asthenia - Nausea - Increased appetite - Dry mouth	

## Data Availability

No new data were created or analyzed in this study. Data sharing is not applicable to this article.
